# Can Intraoperative Anesthesiological Management Reduce the Risk of Acute Kidney Injury After Liver Transplantation? A Systematic Review

**DOI:** 10.3390/jcm15062181

**Published:** 2026-03-12

**Authors:** Filippo Del Tedesco, Giovanni Punzo, Valeria Di Franco, Rita Gaspari, Teresa Sacco, Rikardo Xhemalaj, Tiziana Bove, Paola Aceto

**Affiliations:** 1 Department of Emergency, Anesthesiological and Reanimation Sciences, Fondazione Policlinico Universitario Agostino Gemelli IRCCS, 00168 Rome, Italy; filippo.deltedesco@policlinicogemelli.it (F.D.T.); difrancovaleria@gmail.com (V.D.F.); rita.gaspari@unicatt.it (R.G.); teresa.sacco@policlinicogemelli.it (T.S.); rikardo.xhemalaj@guest.policlinicogemelli.it (R.X.); tiziana.bove@unicatt.it (T.B.); paola.aceto@unicatt.it (P.A.); 2 Department of Basic Biotechnological Sciences, Intensive Care Peri-Operative Clinics, Università Cattolica del Sacro Cuore, 00168 Rome, Italy

**Keywords:** acute kidney injury, postoperative AKI, liver transplantation, anesthesiological management, coagulation profile, liver resection, viscoelastic tests, intraoperative hypotension

## Abstract

**Background:** Acute kidney injury (AKI) is a frequent and severe complication after liver transplantation (LT), occurring in 30–60% of cases. It increases mortality, prolongs hospital stay, and increases the risk of chronic kidney disease. Intraoperative, modifiable anesthetic factors play a key preventive role. This systematic review synthesizes the overall prevalence of AKI and examines the evidence linking intraoperative anesthetic management to AKI after LT, emphasizing modifiable factors that may inform future perioperative strategies. **Methods**: We conducted a systematic, computerized search on PubMed, EMBASE, Cochrane Library, and Scopus from January 2004 to November 16, 2025, following a registered protocol on PROSPERO (ID: CRD420250580749). Randomized controlled trials (RCTs) and cohort studies assessing intraoperative predictors of AKI were considered eligible for inclusion. The primary outcome was the incidence of post-LT AKI. Intraoperative factors associated with post-LT AKI, including intraoperative hypotension, fluid therapy, transfusion strategies, and the use of vasopressors and/or inotropic agents, were also assessed. **Results**: A total of 50 studies (8 RCTs and 42 cohort studies) involving 22,434 patients were included. The pooled incidence of post-LT AKI from observational studies was 41% (95% CI 36–46%). Across the included studies, intraoperative hemodynamic instability, excessive or unbalanced fluid administration, liberal transfusion practices, and suboptimal use of vasopressors were consistently associated with an increased risk of post-transplant AKI. **Conclusions**: AKI after LT is mainly influenced by modifiable perioperative factors. Prevention relies on maintaining stable hemodynamics, careful fluid and transfusion management, and avoiding intraoperative hypotension. Prompt and adequate vasopressor support appeared protective. A multimodal, personalized, kidney-protective approach is essential for improving post-transplant outcomes.

## 1. Introduction

Acute kidney injury (AKI) is a common and clinically significant complication after liver transplantation (LT), with reported incidences ranging from 30% to 60%, depending on the definition used and the population studied [1,2,3]. Post-LT AKI is linked to higher mortality rates, longer intensive care unit (ICU) and hospital stays, and an increased risk of developing chronic kidney disease [4]. The etiology of AKI after LT is multifactorial, encompassing preoperative renal function, a high Model for End-Stage Liver Disease (MELD) score, age, and comorbidities. While many studies have identified pre- and postoperative risk factors, the impact of intraoperative potentially modifiable variables, particularly those related to anesthesiological management, remains less well characterized and may be underappreciated. Intraoperative factors that anesthesiologists can quickly address include hemodynamic instability, the type and volume of fluids administered, the use of vasopressors and/or inotropic agents and anesthetic agents, acid–base balance, and body temperature regulation—all of which can influence renal perfusion [1,2,5,6,7,8,9,10,11,12].

Given the complexity and high risk of LT, understanding the role of modifiable intraoperative anesthetic factors in AKI development is crucial for designing effective prevention strategies and improving patient outcomes.

This systematic review aims to synthesize the overall prevalence of AKI and critically evaluate the existing evidence on how intraoperative anesthetic practices affect the incidence of AKI after liver transplantation, highlighting potentially modifiable determinants and identifying areas for future research.

## 2. Materials and Methods

### 2.1. Search Strategy

We conducted a systematic, computerized search on PubMed, EMBASE, Cochrane Library, and Scopus from January 2004 to 16 November 2025, following a registered protocol on PROSPERO (ID: CRD420250580749) and adhering to the Preferred Reporting Items for Systematic Reviews and Meta-Analyses (PRISMA) guidelines (see Table S1) [13]. The PubMed search, which included MeSH terms, was based on the following main search components: “acute kidney injury,” “liver transplantation,” and “intraoperative predictors.” (“acute kidney injury” [MeSH Terms] OR (“acute” [All Fields] AND “kidney” [All Fields] AND “injury” [All Fields]) OR “acute kidney injury” [All Fields]) AND (“liver transplantation” [MeSH Terms] OR (“liver” [All Fields] AND “transplantation” [All Fields]) OR “liver transplantation” [All Fields]) AND (“intraop” [All Fields] OR “intraoperative” [All Fields] OR “intraoperatively” [All Fields]). The same conceptual search strategy was applied across all selected databases, based on the key domains of liver transplantation, acute kidney injury, and intraoperative/anesthesiologic factors. However, the search syntax was adapted to each database based on its specific indexing system and functionality (see Appendix S1 in the Supplementary Materials).

In addition to database searches, the reference lists of eligible trials and relevant reviews were reviewed to identify any additional studies that met the inclusion criteria. Before final analyses, all searches were repeated to ensure nothing was missed. As outlined in the registered protocol, only preliminary searches had been completed at the time of submission, consistent with PROSPERO policy.

ChatGPT-5.2 (OpenAI, San Francisco, CA, USA) was used exclusively for English language editing and grammatical revision. No part of the study design, data analysis, or interpretation was assisted by the tool.

### 2.2. Study Population

The review study focused on adult patients with end-stage liver disease undergoing LT.

### 2.3. Study Selection

Inclusion criteria were as follows:

(1) randomized controlled trials (RCTs) or cohort studies;
(2) studies evaluating intraoperative predictors of acute kidney injury (AKI);
(3) studies examining either deceased donor liver transplantation (DDLT) or living donor liver transplantation (LDLT);
(4) studies involving patients transplanted for acute liver failure/fulminant hepatitis or chronic liver failure;
(5) studies with any duration of postoperative follow-up.

The exclusion criteria included studies involving pediatric populations, combined liver–kidney and liver transplants, and specific populations (e.g., those with Hepatorenal Syndrome or chronic kidney disease). Non-English studies, study protocols, review articles, editorials, letters, comments, and case reports or case series were excluded.

### 2.4. Outcomes

The primary outcome was the incidence of post-LT AKI. We also evaluated intraoperative factors that influence post-LT AKI, including hemodynamic parameters, e.g., intraoperative hypotension (IOH), fluid therapy, transfusion strategies, use of vasoactive agents, and other modifiable factors.

### 2.5. Data Extraction (Selection and Coding)

Titles and/or abstracts of studies identified through the search strategy and from additional sources were independently screened by two reviewers (F.D.T. and P.A.) to identify studies that potentially met the inclusion criteria outlined above. The full texts of these potentially eligible studies were then retrieved and independently assessed for eligibility by two members of the review team. Any disagreement over the eligibility of specific studies was resolved through discussion, with a third review author involved if necessary (V.D.F.). A standardized form was used to extract data from the included studies. We collected bibliographic details such as the author and year of publication, along with data on the following study characteristics: first author; year of publication; study design; sample size; type of donor; AKI classification; AKI prevalence (primary outcome); other postoperative outcomes; and relevant findings related to intraoperative risk factors for AKI. Two review authors extracted data independently (P.A. and F.D.T.), and discrepancies were identified and resolved through discussion. Data from the studies were recorded on a standardized table developed by the authors. Missing data were requested from the study authors, and if no response was received, a second request was sent. If there was still no response, analyses were conducted using available case data.

### 2.6. Risk of Bias (Quality) Assessment

Once an agreement was reached on the included studies, the two review authors (F.D.T. and V.D.F.) conducted a quality assessment. Each study was evaluated for quality using the bias assessment tools: the Newcastle–Ottawa Scale for cohort studies and the Risk of Bias (ROB) version 1 Cochrane tool for RCTs. Disagreements in the quality assessments were resolved through discussion and, if necessary, with a third reviewer (P.A.).

### 2.7. Strategy for Data Synthesis

The data synthesis is presented in narrative form, examining the predictors identified across the included studies.

### 2.8. Subgroups or Subsets

The different subsets were the various predictors of outcomes identified in the included studies. The data synthesis of the predictors across the included studies was presented narratively.

Intraoperative exposures were detailed and grouped into clinically coherent domains to facilitate structured comparison across studies:

(A) hemodynamic parameters (including hypotension thresholds and duration metrics),
(B) fluid management (crystalloids, colloids, balanced vs. non-balanced solutions, cumulative volumes),
(C) transfusion strategy (type and amount of blood products, reported transfusion triggers),
(D) vasoactive drug use (agent type, cumulative dose, and timing when available),
(E) Other modifiable factors.

Given the substantial variability in exposure definitions and measurement approaches across studies, quantitative pooling of effect estimates was deemed methodologically inappropriate, and a structured qualitative synthesis was conducted instead.

### 2.9. Statistical Analysis

A meta-analysis of raw proportions (PRAW) was conducted to estimate the overall incidence of AKI after liver transplantation, using only observational studies.

Post hoc subgroup analyses were conducted using the same random-effects model of raw proportions to explore potential differences by transplantation type (LDLT vs. DDLT), AKI definition (KDIGO vs. non-KDIGO), and study era (2004–2014 vs. 2015–2025). Differences between subgroups were assessed using the Q-test within the random-effects framework.

Small-study effects were evaluated using Egger’s regression test based on a linear regression of the effect size on its standard error.

The analysis was performed using R software version 4.5.2 (R Foundation for Statistical Computing, Vienna, Austria) using the meta and metafor packages.

Proportions were pooled using a random-effects model (REML method), and the 95% confidence interval, prediction interval, and heterogeneity statistics (I^2^ and τ^2^) were calculated.

## 3. Results

### 3.1. Selection and Characterization of the Selected Studies

**Figure 1:**
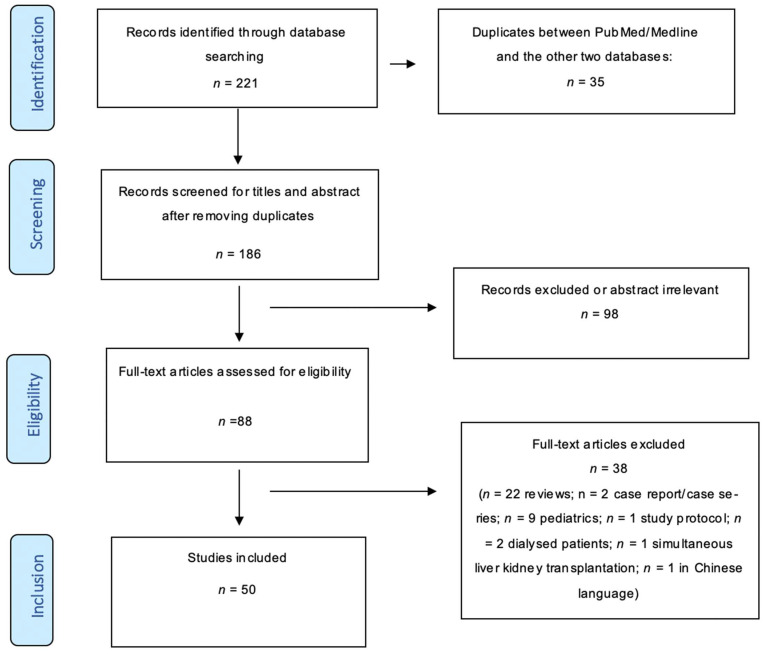
PRISMA Flow Diagram. The diagram reflects the results of the final literature search conducted on 16 November 2025.

After applying inclusion and exclusion criteria, 50 articles (8 RCTs and 42 cohort studies) were included, encompassing 22,434 patients. The PRISMA flowchart is shown in Figure 1, and Supplementary Table S1 (see Supplementary Materials) summarizes the characteristics of each study.

Most studies (*n* = 28) used the KDIGO (Kidney Disease: Improving Global Outcomes) definition of AKI, while eight employed the RIFLE (risk, injury, failure, loss, and end-stage kidney disease) criteria, one used the RIFLE-AKIN (Acute Kidney Injury Network) criteria, two applied ICA (International Club of Ascites), one used the AKIN criteria, and another used ICA and ADQI (Acute Disease Quality Initiative) criteria. The remaining studies did not use standardized AKI definitions.

### 3.2. Primary Outcome

**Figure 2:**
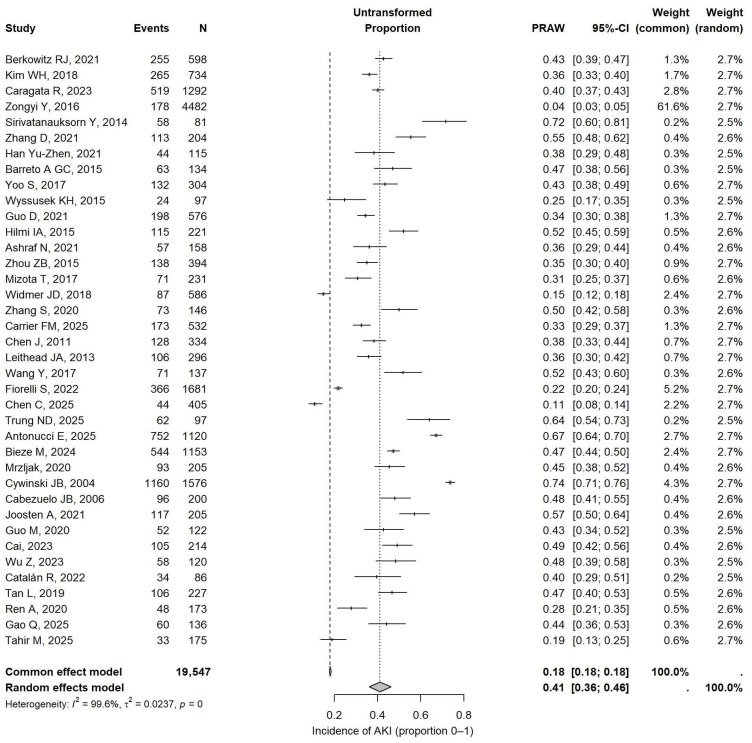
Forest plots reporting the prevalence of AKI across cohort studies. The effect size is calculated as the untransformed proportion (0–1) and the corresponding 95% confidence interval (95%CI). Studies included in the present figure: Berkowitz RJ, 2021 [2]; Kim WH, 2018 [14]; Caragata R, 2023 [3]; Zongyi Y, 2016 [5]; Sirivatanauksorn Y, 2014 [4]; Zhang D, 2016 [15]; Han Yu-Zhen, 2021 [10]; Barreto A GC, 2015 [6]; Yoo S, 2017 [11]; Wissusek KH, 2015 [16]; Guo D, 2021 [17]; Hilmi IA, 2015 [18]; Ashraf N, 2021 [19]; Zhou ZB, 2015 [20]; Mizota T, 2017 [21]; Widmer JD, 2018 [22]; Zhang S, 2020 [12]; Carrier FM, 2025 [23]; Chen J, 2011 [24]; Leithead JA, 2013 [25]; Wang Y, 2017 [26]; Fiorelli S, 2022 [1]; Chen C, 2025 [7]; Trung ND, 2025 [27]; Antonucci E, 2025 [28]; Bieze M,204 [29]; Mrzljak, 2020 [30]; Cywinski JB, 2004 [31]; Cabazuelo JB, 2006 [32]; Joosten A, 2021 [33]; Guo M, 2020 [34]; Cai, 2023 [35]; Wu Z, 2023 [36]; Catalàn R, 2022 [37]; Tan L, 2019 [38]; Ren A, 2020 [39]; Gao Q, 2025 [40]; Tahir M, 2025 [41].

Among the included observational studies, the pooled incidence of AKI after LT, expressed as an untransformed proportion, was 0.41 (95% CI 0.36–0.46), indicating that about 41% of patients developed postoperative AKI (Figure 2).

Subgroup analyses stratified by transplantation type, AKI definition, and study era did not show statistically significant differences between groups. The Q-test for subgroup differences was non-significant across all comparisons (see Supplementary Figures S1–S3 in the Supplementary Materials).

### 3.3. Secondary Outcomes

The following Section 3.3.1, Section 3.3.2, Section 3.3.3, Section 3.3.4 and Section 3.3.5 summarize intraoperative risk factors for post-LT AKI identified in the literature.

#### 3.3.1. Hemodynamic Parameters

Baseline elevated central venous pressure (CVP), reflecting venous congestion and renal hypoperfusion, and reduced mixed venous oxygen saturation (SvO_2_), indicating inadequate oxygen delivery, before reperfusion of the new liver, were identified as independent predictors of AKI after LDLT [14]. A retrospective two-center study comparing low versus normal CVP strategies during LT found that while low CVP significantly reduced blood transfusion requirements, it was associated with higher peak creatinine levels, increased postoperative dialysis, and greater 30-day mortality. The authors conclude that intentional hypovolemia to lower CVP is unsafe and should not be used [42]. Seven retrospective studies focusing on IOH included both LDLT and DDLT, with sample sizes ranging from 81 to 1576 patients [3,4,16,21,29,31,33]. Definitions of IOH varied across these studies (Table 1); six studies used a mean arterial pressure (MAP) between 55 and 70 mmHg as the primary or combined threshold [3,4,21,29,31,33], while one study used systolic blood pressure (SBP) < 90 mmHg as the primary threshold [16]. All studies also considered the duration of hypotension (from 15 to 30 min). AKI was defined according to KDIGO in five studies [3,4,29,31,33] and RIFLE in two studies [16,21]. Four studies demonstrated an association between IOH and AKI [3,4,21,33], while one study reported a strong link between post-reperfusion hypotension and AKI [16]. In contrast, two studies found no significant relationship between IOH and post-LT AKI [16,31].

#### 3.3.2. Fluid Management

##### Crystalloids

In the largest LDLT cohort study (*n* = 1011), the intraoperative use of 0.9% saline was associated with a higher AKI rate than balanced crystalloids (50.6% vs. 38.5%; *p* = 0.010) [43]. Similarly, Nadeem et al. (2014) (*n* = 158) found that a chloride load >3.2 L/24 h—mainly from saline and saline-diluted albumin—independently predicted AKI (Hazard Ratio 6.25, 95% CI 2.69–14.5) [19]. Both studies indicate that chloride-rich fluids increase postoperative renal risk compared with balanced crystalloids [19,43].

##### Colloids

In a retrospective cohort study (Hand et al., 2015; *n* = 174), exposure to hydroxyethyl starch (HES) was associated with nearly a threefold increased risk of AKI compared to 5% albumin (adjusted OR, 2.94; 95% CI, 1.13–7.7) [44]. Conversely, in a study involving 394 liver transplant recipients, there was no significant difference in postoperative renal function associated with intraoperative HES use (200/0.5 or 130/0.4), suggesting short-term renal safety in LT [20]. In a randomized trial involving 40 LDLT recipients, HES 130/0.4 and 5% albumin showed similar renal outcomes, with no differences in creatinine, creatinine clearance, or cystatin C. Although the HES group received higher fluid volumes and had a greater cumulative balance, AKI rates were equivalent between groups [45].

##### Fluid Balance

Zhang et al. (2020) (*n* = 146) reported that a positive fluid balance exceeding 5 L within 72 h doubled the risk of AKI (53% vs. 28%; OR 2.11, 95% CI 1.22–3.64) [12]. Similarly, Guo et al. (2021) (*n* = 576) [17] and Mrzljak et al. (2020) (*n* = 205) [30] identified greater intraoperative positive balance and total fluid intake, respectively, as predictors of severe AKI. Carrier et al. (2020) (*n* = 532) found that fluid balance was unrelated to AKI but associated with longer ICU stays and lower survival rates [9]. Overall, these findings suggest that both hypovolemia and excessive fluid accumulation negatively affect renal outcomes.

#### 3.3.3. Transfusion Strategy

Transfusion volume and product characteristics are consistently associated with postoperative AKI [6,25,36,38,41]. In Barreto et al. (2015) (*n* = 200) [6], as well as in Tan et al.’s study (*n* = 227) [38], transfusion of ≥10 red blood cell (RBC) units significantly increased the incidence of AKI. Hannon et al. (2020) (*n* = 500) demonstrated a dose–response relationship between RBC transfusion volume and AKI as defined by KDIGO criteria [46].

Hilmi et al. (2015) (*n* ≈ 300) confirmed that transfusion requirements are an independent predictor of AKI, even after adjusting for hemodynamic instability [18]. Wang et al. (2017) (*n* = 283) reported that transfusion of old RBCs stored for ≥14 days increased the risk of both AKI (42.5% vs. 28.9%; OR 1.82, 95% CI 1.05–3.15) and severe AKI (KDIGO 3) (OR 2.11, 95% CI 1.13–3.97) [26]. Guo M. et al. (2021) (*n* = 576) found that intraoperative fresh frozen plasma (FFP) volume was a strong predictor of severe AKI (OR 1.34, 95% CI 1.03–1.75 per 1000 mL) [34].

In a large multicenter cohort (*n* = 1681), both RBC and FFP transfusions independently predicted postoperative AKI after adjusting for confounders [1]. Three studies (Tahir, 2025; Cai, 2023; and Ren, 2020) identified estimated blood loss as a risk factor for AKI [35,39,41]. Additionally, Trung et al. (2025) (*n* = 97 LDLT) found blood loss (OR 1.81, 95% CI 1.05–3.09) and early postoperative lactate (OR 1.61, 95% CI 1.21–2.15) to be independent predictors of early AKI [27].

Chen et al. (2025) proposed the Surgical–Anhepatic–Liver Transplantation (SALT) nomogram for severe AKI, incorporating the MELD score, blood loss, ALT, D-dimer, and viscoelastic parameters [24]. The model showed good discrimination (AUC 0.81; 95% CI 0.76–0.85) and calibration across both living and deceased donor LT groups, confirming the substantial predictive value of intraoperative hemodynamic instability and transfusion burden for postoperative renal dysfunction [24]. Overall, evidence indicates that high chloride exposure, HES use, excessive fluid or transfusion volumes, and older blood products are consistently linked to an increased risk of AKI. In contrast, balanced crystalloids and albumin-based resuscitation are linked to improved renal outcomes.

#### 3.3.4. Vasoactive Drug Use

In a small RCT, fenoldopam proved more effective than dopamine at maintaining creatinine clearance, whereas dopamine was linked to a decline of up to 39% compared with placebo [47]. However, this result was not confirmed in a subsequent RCT [48]. In a cohort of 184 LT recipients, Cabezuelo et al. identified the duration of postoperative dopamine infusion as an independent risk factor for AKI (OR 1.6 per day, 95% CI 1.3–2.1), indicating that dopamine exposure may reflect underlying hemodynamic instability rather than provide renal protection [32].

This interpretation was later confirmed by multicenter prospective evidence. The LT study by Fiorelli et al. (*n* = 1681) demonstrated that intraoperative dopamine exposure independently predicted postoperative AKI (OR 1.6, 95% CI 1.2–2.3), even after adjusting for transfusion requirements, reperfusion syndrome, and surgical complexity [1].

Observational studies have linked norepinephrine use to postoperative AKI, although most results are confounded by hemodynamic instability. In the cohort study by Cabezuelo et al. (*n* = 184), norepinephrine was more commonly used among AKI cases, reflecting perioperative instability rather than direct nephrotoxicity [32]. Chen et al. (2011) (*n* = 384) also found that intraoperative vasopressor use—primarily norepinephrine—was independently linked to AKI, noting confounding by indication [7]. In an Australian cohort, Wyssusek et al. (*n* = 97) confirmed this association and reported higher BMI and MELD scores, again interpreting norepinephrine need as a severity marker [16].

In a multicenter study (*n* = 1681), postoperative norepinephrine infusion was associated with AKI (OR 1.4, 95% CI 1.0–2.0) after adjustment for transfusion and surgical factors [1]. Conversely, Bieze et al. (*n* = 1153) reported that maintaining MAP > 70–75 mmHg with prompt vasopressor titration (mainly norepinephrine) was associated with reduced rates of moderate-to-severe AKI [29].

Among non-catecholaminergic vasoconstrictors, terlipressin infusion was evaluated in 50 patients undergoing LDLT. The incidence of AKI did not differ between the terlipressin and control groups (44% vs. 48%) [49]. However, norepinephrine requirements were significantly lower in the terlipressin group, suggesting reduced catecholamine demand without renal benefit [49]. In a recent study (Antonucci et al., 2025, *n* = 1120), intraoperative vasopressin use was not linked to severe AKI or graft failure, indicating its safety for hemodynamic support without increasing postoperative renal risk [28].

#### 3.3.5. Other Modifiable Factors

In a randomized controlled trial, intraoperative dexmedetomidine infusion reduced postoperative AKI in LDLT recipients compared with standard care (35.0% vs. 50.0%; *p* = 0.042) [50]. Berkowitz et al. reported that intraoperative increases in serum potassium and lactate were independent predictors of AKI (adjusted OR for potassium: 1.36; 95% CI 1.12–1.66) [2]. Two studies confirmed these findings. Specifically, Catalan (2022) identified high lactate concentrations during surgery (7.2 mmol/L ± 3.3) [37], while Gao Q. et al., 2025, found elevated anhepatic potassium levels to be an independent and strong predictor of postoperative AKI in multivariate analysis [40]. Other studies confirmed that preoperative metabolic acidosis and electrolyte imbalances are strong predictors of AKI, although sodium bicarbonate infusion did not significantly reduce the incidence of AKI [51]. In a cohort of 84 LDLT recipients, pre-reperfusion mannitol (1 g/kg) did not reduce early AKI or post-reperfusion syndrome. It did not provide intraoperative hemodynamic benefit compared with saline [52].

Procedural factors such as cold ischemia time, total surgery duration, and duration of inferior vena cava clamping can influence outcomes and may offer opportunities to prevent AKI in liver transplantation [5,15,29,46]. Recent evidence supports surgical techniques that reduce warm ischemia time to help preserve renal function [5,6,36]. Studies on liver transplantation techniques reported conflicting renal outcomes. The piggyback technique appears to reduce the risk of severe AKI compared with caval replacement [36], whereas the conventional approach with venovenous bypass is associated with increased postoperative renal dysfunction [53]. Notably, caval replacement without bypass yields outcomes comparable to those of the piggyback technique [22].

### 3.4. Risk of Bias

**Figure 3:**
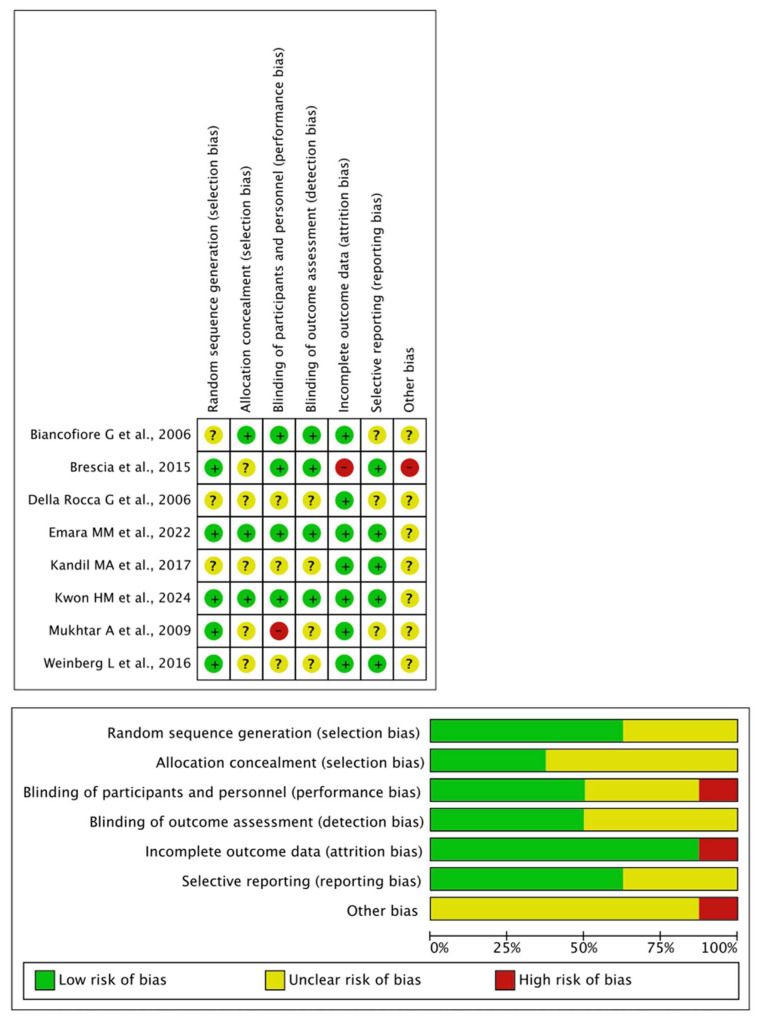
Risk of bias of RCT. Green (+): Low risk of bias; Yellow (?): unclear risk of bias; Red (−): high risk of bias. RCT included in the present figure: Biancofore G et al. [48]; Brescia et al. [53]; Della Rocca G et al. [47]; Emara MM et al. [52]; Kandil MA et al. [49]; Kwon HM et al. [50]; Mukhtar A et al. [45]; Weinberg L et al. [51].

Most cohort studies provided adequate outcome assessment and follow-up; however, comparability across groups, particularly with respect to gender and MELD score, was often limited. Some studies lacked clear representativeness of the exposed cohort or did not adequately report control selection criteria (see Supplementary Table S2). In randomized trials, reporting on randomization and allocation concealment was often incomplete, and blinding of personnel was generally not feasible in the surgical setting (see Figure 3).

Visual inspection of the funnel plot did not reveal marked asymmetry, and Egger’s regression test did not provide statistically significant evidence of small-study effects (z = 0.66, *p* = 0.51) (see Figure S4 in the Supplementary Materials).

## 4. Discussion

Meta-analyses of the included cohorts reported a pooled incidence of acute kidney injury (AKI) of 0.40 (95% CI 0.34–0.46) after liver LT, confirming that postoperative AKI remains a significant clinical burden, affecting nearly two-fifths of recipients. Current evidence suggests that AKI after LT is a complex but largely preventable event rather than an inevitable consequence of advanced liver disease. Intraoperative hemodynamic management, fluid strategies, transfusion exposure, and vasopressor use consistently emerge as key modifiable determinants of renal outcomes. However, most available studies are retrospective and single-center, limiting causal inference and leaving key points, such as optimal MAP targets, ideal fluid composition, and transfusion thresholds unanswered.

Intraoperative hypotension, particularly sustained reductions in MAP, is a well-established contributor to renal injury. Renal blood flow is typically autoregulated within a MAP range of 50–60 mmHg; below this threshold, perfusion becomes pressure-dependent, increasing the risk of ischemic and tubular damage. During the anhepatic and reperfusion phases, hemodynamic instability, reduced venous return, and systemic inflammatory responses significantly impair renal perfusion [54]. When combined with portal hypertension and blood loss, these factors create a physiological environment that promotes ischemic injury [54]. Observational data consistently link MAP below 60–65 mmHg with increased AKI rates, supporting perioperative strategies to keep MAP above this threshold through goal-directed fluid therapy and careful bleeding control [3,4,21,29,33].

In LTs, ischemia–reperfusion injury is a key factor that affects both transplant outcome and the development of postoperative complications such as AKI. Post-reperfusion syndrome—an immediate drop in mean arterial pressure within minutes of reperfusion—serves as an early clinical indicator [1,2,22,29,42]. This hemodynamic instability, driven by vasodilation, hypovolemia, and impaired vasoconstrictive responses, further reduces renal perfusion and increases susceptibility to AKI [29,54]. Preventive strategies, including surgical techniques (e.g., piggyback), anesthetic interventions (e.g., dexmedetomidine), and organ perfusion technologies, have demonstrated benefits in attenuating hemodynamic instability, reducing post-reperfusion syndrome, and lowering the incidence of postoperative AKI [22,46,50,53,54]. Post-reperfusion syndrome, higher transfusion volumes, and increased vasopressor requirements are often linked to intraoperative hypotension and postoperative AKI, indicating potential confounding effects among these interconnected factors [1,54].

Fluid administration is another modifiable determinant of renal outcomes. Both fluid type and chloride content influence postoperative kidney function. Normal saline is associated with higher AKI rates than balanced crystalloids [43], especially when large chloride loads are delivered [19]. Hyperchloremic metabolic acidosis, which induces afferent arteriolar vasoconstriction and reduces cortical perfusion, is central to this effect [43]. These findings support the preferential use of balanced crystalloids and a chloride-aware resuscitation approach during intraoperative care [19,43]. Regarding colloids, although HES has not demonstrated clear nephrotoxicity in LT [20,45], albumin remains the preferred agent because of its favorable renal safety profile, maintenance of oncotic pressure, and ability to bind circulating toxins [20,44,45].

Perioperative fluid balance also critically affects renal outcomes. Excessive positive fluid balance in the early postoperative period independently predicts AKI and the need for renal replacement therapy [19]. Intraoperative fluid overload and plasma transfusion further increase risk. Fluid accumulation promotes renal venous congestion and raises interstitial pressure, reducing filtration gradients and exacerbating hyperchloremic acidosis when chloride-rich fluids are used [19]. Modeling data confirm that cumulative blood loss and metabolic stress are independent predictors of AKI, underscoring the importance of minimizing bleeding, optimizing transfusion thresholds, and mitigating metabolic derangements during LT [24,27]. Fluid and transfusion exposure contribute to AKI through a venous congestion pathway [24]. These observations support the use of tailored, goal-directed strategies to maintain euvolemia, avoiding both under-resuscitation and fluid overload. Although LT-specific protocols are lacking, advanced hemodynamic monitoring may enhance renal protection [55].

Transfusion significantly influences renal outcomes. Several studies report a dose–response relationship between intraoperative red blood cell and plasma transfusion volumes and AKI risk [1,5,16,25,26,36,38,41]. Transfusion of older red blood cell units (≥14 days) nearly doubles the risk of postoperative AKI, likely due to storage-related changes that impair microcirculatory flow [26]. The Vietnamese LDLT cohort [27] and Surgical–Anhepatic–Liver Transplantation [24] identified blood loss and metabolic stress as independent predictors of AKI, indicating that transfusion exposure reflects both therapeutic decisions and surgical severity.

Transfusion-related renal injury is multifactorial, driven by transfusion volume, product quality, and bleeding severity, and is further exacerbated by fluid overload and vasopressor requirements [2]. Restrictive, goal-directed transfusion strategies are recommended, prioritizing fresher RBCs [26], judicious plasma use, and substituting targeted coagulation factor concentrates where appropriate [56]. Viscoelastic testing (ROTEM, TEG) enables patient-specific, real-time assessment of coagulation, reducing unnecessary product use, preventing volume overload, and lowering AKI risk [57,58].

The relationship between vasopressors and AKI is confounded mainly by indication, reflecting intraoperative instability rather than direct nephrotoxicity. Patients requiring catecholamine support for hypotension, post-reperfusion syndrome, or hemorrhage are inherently at higher risk. Although vasopressor use often appears as an independent predictor of AKI, causality is unlikely [1,7,9,32,48,49]. Prospective and randomized studies failed to show any kidney benefit from “renal-dose” dopamine (1–3 µg/kg/min), which was commonly used in liver transplant anesthesia and postoperative care in the 1990s–2000s; low-dose dopamine provides no kidney protection and may even worsen outcomes [32,48]. Terlipressin reduces norepinephrine requirements but does not offer renal protection once autoregulation fails [49]. Norepinephrine—used early and titrated—emerges as a safer and more effective vasopressor for supporting renal perfusion in this setting, effectively restoring perfusion pressure without compromising renal oxygenation [59]. In a recent systematic review and network meta-analysis, Carrier F.M. et al. demonstrated that none of the intraoperative vasoactive drugs improved AKI [23]. Finally, Jan et al., in a comprehensive review of over 40 studies, emphasized that vasopressor exposure—particularly norepinephrine and dopamine—is consistently linked to AKI after LT, with reported ORs ranging from 1.4 to 2.4 across large groups [60]. Notably, the authors emphasized that this reflects vasopressor use as a sign of instability, post-reperfusion syndrome, or hemorrhage, rather than a direct nephrotoxic effect. The review concluded that the risk of kidney damage related to vasopressors depends on the context (hemodynamic status and patients characteristics) and dose [60].

Among anesthetic agents, dexmedetomidine may offer renal protection through sympatholytic and anti-inflammatory effects [50], though evidence is limited. Acid–base disturbances, especially metabolic acidosis and hyperkalemia, are consistently associated with AKI due to impaired perfusion and tissue hypoxia [2]. Sodium bicarbonate has not been shown to reduce AKI and should be used only in oligoanuric patients to treat hyperkalemia [40,51]. Although intraoperative temperature management has not been directly associated with AKI, hypothermia causes renal vasoconstriction and oxidative stress, which could be areas for future research.

This review has several limitations. Most included studies were retrospective and single-center, which limited causal inference and increased susceptibility to selection bias and unmeasured confounding. In this meta-analysis, substantial between-study heterogeneity was observed, likely reflecting differences in study design and clinical characteristics. AKI definitions (KDIGO, RIFLE, ICA, or non-standard criteria) and assessment time points (48 h, 72 h, or 7 days) varied widely, complicating direct comparisons across cohorts and partly explaining the wide range of reported incidence estimates.

The relative proportions of donation after brain death (DBD) and donation after circulatory death (DCD) donors varied widely across the selected reports, and in some studies, the type of deceased donor liver transplanted was not clearly specified. This variability may have influenced the reported rates of reperfusion syndrome and potentially acute kidney injury (AKI) observed among recipients across the included studies, particularly those receiving DCD livers. Additionally, it remains unclear how individual liver transplant programs determine which subtype of deceased donor liver is allocated to specific recipient profiles, further confounding the results of this systematic review.

Although subgroup analyses by publication period, donor type, and AKI definition were conducted to explore potential sources of heterogeneity, variability remained high, suggesting that additional unmeasured methodological and clinical factors contribute to the observed dispersion. Although meta-regression can be useful for exploring heterogeneity, its application in this context would have been methodologically fragile because of extreme residual heterogeneity, small numbers of studies per covariate, and reliance on aggregate study-level data. These factors increase the risk of unstable estimates and ecological bias. Therefore, subgroup analyses were preferred as a more robust and clinically interpretable exploratory approach.

Visual inspection of the funnel plot did not reveal marked asymmetry, and Egger’s regression test was not statistically significant, indicating no strong statistical evidence of small-study effects. Nevertheless, given the considerable heterogeneity, publication bias assessments should be interpreted cautiously.

A major limitation is the marked heterogeneity in how intraoperative exposures were defined and measured. Key intraoperative parameters—mean arterial pressure thresholds, vasopressor dosing, transfusion triggers, and fluid composition—were inconsistently documented, hindering identification of optimal hemodynamic or resuscitation targets. Factors critical to renal injury, including graft quality, donor stability, technical complexity, and ischemia–reperfusion severity, were often inconsistently reported and seldom adjusted for in multivariate analyses. Evidence regarding temperature management, acid–base correction, chloride load, and real-time goal-directed monitoring was limited despite their physiological importance. Vasopressor-related findings were particularly difficult to interpret because of strong confounding by indication, as patients receiving catecholamines were inherently more unstable. Many studies did not adequately adjust for baseline hemodynamic instability or intraoperative severity, and inconsistencies in vasopressor selection, dosing strategies, and timing further limited standardized comparisons. Studies evaluating perfusion technologies, postoperative fluid balance, and metabolic complications lacked standardized methodology and follow-up. Overall, the predominance of observational data limits the ability to draw firm causal inferences with post-LT AKI.

Collectively, these methodological inconsistencies likely influenced reported associations and prevented a robust quantitative synthesis of risk estimates, underscoring the need for standardized exposure definitions and rigorously designed prospective studies to enhance comparability and enable reliable pooled analyses.

## 5. Conclusions

In summary, post-LT AKI appears to be associated with intraoperative hemodynamic instability, fluid and transfusion management, and metabolic disturbances. Maintaining hemodynamic stability, minimizing prolonged hypotension, and adopting individualized perfusion strategies may be modifiable factors; however, current evidence is predominantly observational and does not support firm causal conclusions.

Associations reported across studies suggest that avoiding sustained intraoperative hypotension, using goal-directed fluid strategies, and carefully titrating vasopressors and transfusion practices could be relevant components of perioperative management. Nonetheless, heterogeneity in exposure definitions and methodological limitations preclude definitive recommendations on specific MAP thresholds, fluid composition, or vasopressor strategies.

Large, adequately powered, multicenter randomized trials are urgently needed to establish evidence-based intraoperative targets and to determine whether integrated hemodynamic, metabolic, and transfusion strategies can reduce the incidence of AKI after liver transplantation.

## Figures and Tables

**Table 1:** Summary of Included Studies Evaluating IOH and AKI.

Study	Design/*n*	Graft Type	IOH Definition	AKI Criteria	Main Findings (Adjusted)
Sirivatanauksorn Y. et al., 2014 [4]	Retrospective *n* = 81	DDLT	MAP < 70 mmHg	KDIGO	Prolonged (>30 min) IOH
Wyssusek K.H. et al., 2015 [16]	Retrospective *n* = 97	Mixed	SBP < 90 mmHg for >15 min	RIFLE	IOH was not associated with AKI
Mizota T. et al., 2017 [21]	Retrospective *n* = 231	LDLT	MAP < 55 & <65, cumulative duration	RIFLE	MAP < 55 mm Hg >20 min → severe AKI
Joosten A. et al., 2021 [33]	Retrospective *n* = 205	DDLT	Time-weighted (MAP) < 65 mmHg	KDIGO	Prolonged (Quartile 4, >39.5%) IOH
Caragata R. et al., 2023 [3]	Retrospective *n* = 1292	Mixed	MAP < 55 mmHg	KDIGO	Prolonged (>20 min) IOH
Bieze et al., 2024 [29]	Retrospective *n* = 1153	DDLT	MAP < 60 & 55 by the surgical phase	KDIGO	Reperfusion-phase IOH is the strongest predictor
Cywinski J.B. et al., 2024 [31]	Retrospective *n* = 1576	Mixed	AUT; TWA; time of MAP (<55, 60 and 65 mmHg);	KDIGO	No significant link was found between hypotension exposure and postoperative AKI

MAP, mean arterial pressure; SBP, systolic blood pressure; LDLT, living donor liver transplantation; DDLT, deceased donor liver transplantation; RIFLE; KDIGO, Kidney Disease: Improving Global Outcomes; IOH, intraoperative hypotension; AKI, acute kidney injury; RIFLE (Risk, Injury, Failure, Loss, End-stage kidney disease); AUT: Area under the threshold; TWA: Time-weighted average.

## Data Availability

No new data were created or analyzed in this study. Data sharing is not applicable to this article.

## Supplementary Materials

The following supporting information can be downloaded at: https://www.mdpi.com/article/10.3390/jcm15062181/s1. Appendix S1: Detailed Search Strategy (January 2004–16 November 2025); Table S1: Characteristics of the included studies; Table S2: Risk of bias for assessing the quality of cohort studies by using the Newcastle-Ottawa Scale; Figure S1: Forest plot of pooled AKI incidence by cohort DDLT vs cohort LDLT; Figure S2: Forest plot of pooled AKI incidence by cohort KDIGO vs. cohort no KDIGO; Figure S3: Forest plot of pooled AKI incidence by cohort 2015–2025 vs. cohort 2004–2014; Figure S4: Funnel plot of AKI incidence.

## References

[1] Fiorelli S., Biancofiore G., Feltracco P., Lavezzo B., De Gasperi A., Pompei L., Masiero L., Testa S., Ricci A., Della Rocca G. (2022). Acute kidney injury after liver transplantation, perioperative risk factors, and outcome: Prospective observational study of 1681 patients (OLTx Study). Minerva Anestesiol..

[2] Berkowitz R.J., Engoren M.C., Mentz G., Sharma P., Kumar S.S., Davis R., Kheterpal S., Sonnenday C.J., Douville N.J. (2022). Intraoperative risk factors of acute kidney injury after liver transplantation. Liver Transpl..

[3] Caragata R., Emerson S., Santema M.L., Selzner N., Sapisochin G., Wang S., Huszti E., Van Klei W., McCluskey S.A. (2023). Intraoperative hypotension and the risk of acute kidney injury following liver transplantation. Clin. Transplant..

[4] Sirivatanauksorn Y., Parakonthun T., Premasathian N., Limsrichamrern S., Mahawithitwong P., Kositamongkol P., Tovikkai C., Asavakarn S. (2014). Renal dysfunction after orthotopic liver transplantation. Transplant. Proc..

[5] Zongyi Y., Baifeng L., Funian Z., Hao L., Xin W. (2017). Risk factors of acute kidney injury after orthotopic liver transplantation in China. Sci. Rep..

[6] Barreto A.G., Daher E.F., Silva Junior G.B., Garcia J.H., Magalhães C.B., Lima J.M., Viana C.F., Pereira E.D. (2015). Risk factors for acute kidney injury and 30-day mortality after liver transplantation. Ann. Hepatol..

[7] Chen J., Singhapricha T., Hu K.Q., Hong J.C., Steadman R.H., Busuttil R.W., Xia V.W. (2011). Postliver transplant acute renal injury and failure by the RIFLE criteria in patients with normal pretransplant serum creatinine concentrations: A matched study. Transplantation.

[8] Hall T.H., Dhir A. (2013). Anesthesia for liver transplantation. Semin. Cardiothorac. Vasc. Anesth..

[9] Carrier F.M., Chassé M., Wang H.T., Aslanian P., Iorio S., Bilodeau M., Turgeon A.F. (2020). Restrictive fluid management strategies and outcomes in liver transplantation: A systematic review. Can. J. Anaesth..

[10] Han Y.Z., Zhang J., Gao R.Y., Zhao S., Zheng Y., Ding Q., Xin X., Yu K., Huang L.F., Li W.X. (2021). Predictive utility of postoperative serum myoglobin in acute kidney injury after liver transplantation. Ann. Palliat. Med..

[11] Yoo S., Lee H.J., Lee H., Ryu H.G. (2017). Association between perioperative hyperglycemia or glucose variability and postoperative acute kidney injury after liver transplantation: A retrospective observational study. Anesth. Analg..

[12] Zhang S., Ma J., An R., Liu L., Li J., Fang Z., Wang Q., Ma Q., Shen X. (2020). Effect of cumulative fluid balance on acute kidney injury and patient outcomes after orthotopic liver transplantation: A retrospective cohort study. Nephrology.

[13] Page M.J., McKenzie J.E., Bossuyt P.M., Boutron I., Hoffmann T.C., Mulrow C.D., Shamseer L., Tetzlaff J.M., Akl E.A., Brennan S.E. (2021). The PRISMA 2020 statement: An updated guideline for reporting systematic reviews. BMJ.

[14] Kim W.H., Oh H.W., Yang S.M., Yu J.H., Lee H.-C., Jung C.-W., Suh K.-S., Lee K.H. (2019). Intraoperative hemodynamic parameters and acute kidney injury after living donor liver transplantation. Transplantation.

[15] Zhou D., Liu Z., Bi J.F., Liu H., Gao Y. (2021). Risk factors for the incidence and severity of acute kidney injury after liver transplantation. Turk. J. Gastroenterol..

[16] Wyssusek K.H., Keys A.L., Yung J., Moloney E.T., Sivalingam P., Paul S.K. (2015). Evaluation of perioperative predictors of acute kidney injury post orthotopic liver transplantation. Anaesth. Intensive Care.

[17] Guo D., Wang H., Lai X., Li J., Xie D., Zhen L., Jiang C., Li M., Liu X. (2021). Development and validation of a nomogram for predicting acute kidney injury after orthotopic liver transplantation. Ren. Fail..

[18] Hilmi I.A., Damian D., Al-Khafaji A., Sakai T., Donaldson J., Winger D.G., Kellum J.A. (2015). Acute kidney injury after orthotopic liver transplantation using living donor versus deceased donor grafts: A propensity score-matched analysis. Liver Transpl..

[19] Nadeem A., Salahuddin N., El Hazmi A., Joseph M., Bohlega B., Sallam H., Sheikh Y., Broering D. (2014). Chloride-liberal fluids are associated with acute kidney injury after liver transplantation. Crit. Care.

[20] Zhou Z.B., Shao X.X., Yang X.Y., Zhang T., Xian D.F., Huang C.Y., Yang L., Huang W.Q. (2015). Influence of hydroxyethyl starch on renal function after orthotopic liver transplantation. Transplant. Proc..

[21] Mizota T., Hamada M., Matsukawa S., Seo H., Tanaka T., Segawa H. (2017). Relationship between intraoperative hypotension and acute kidney injury after living donor liver transplantation: A retrospective analysis. J. Cardiothorac. Vasc. Anesth..

[22] Widmer J.D., Schlegel A., Ghazaly M., Richie Davidson B., Imber C., Sharma D., Malago M., Pollok J.M. (2018). Piggyback or cava replacement: Which implantation technique protects liver recipients from acute kidney injury and complications?. Liver Transpl..

[23] Carrier F.M., Girard M., Zuo R.M., Ziegler D., Trottier H., Chassé M. (2024). Intraoperative vasoactive medications and perioperative outcomes in liver transplantation: A systematic review and network meta-analyses. Transplantation.

[24] Chen C., Chen X., Gao Y., Deng Y., Li Z. (2025). Graded nomograms based on perioperative parameters for predicting new-onset severe acute kidney injury following liver transplantation in patients with normal preoperative renal function: The SALT scale. Ren. Fail..

[25] Leithead J.A., Armstrong M.J., Corbett C., Andrew M., Kothari C., Gunson B.K., Muiesan P., Ferguson J.W. (2013). Hepatic ischemia reperfusion injury is associated with acute kidney injury following donation after brain death liver transplantation. Transpl. Int..

[26] Wang Y., Li Q., Ma T., Liu X., Wang B., Wu Z., Dang S., Lv Y., Wu R. (2018). Transfusion of older red blood cells increases the risk of acute kidney injury after orthotopic liver transplantation: A propensity score analysis. Anesth. Analg..

[27] Trung N.D., Van Nam D., Thu N.T., Nam H., Su H.X. (2025). Perioperative risk factors for early acute kidney injury after living donor liver transplantation: A single transplant center in Vietnam. Transplant. Proc..

[28] Antonucci E., Bokoch M.P., Adelmann D., Kolodzie K., Roll G.R., Sun E., Legrand M., Kothari R. (2025). Vasopressin is not associated with severe kidney injury in liver transplantation: A propensity score-adjusted analysis. Transplant. Direct.

[29] Bieze M., Zabida A., Martinelli E.S., Caragata R., Wang S., Carroll J., Selzner M., McCluskey S.A. (2024). Intraoperative hypotension during critical phases of liver transplantation and its impact on acute kidney injury: A retrospective cohort study. Braz. J. Anesthesiol..

[30] Mrzljak A., Franusic L., Pavicic-Saric J., Kelava T., Jurekovic Z., Kocman B., Mikulic D., Budimir-Bekan I., Knotek M. (2020). Pre- and intraoperative predictors of acute kidney injury after liver transplantation. World J. Clin. Cases.

[31] Cywinski J.B., Li Y., Liu X., Khanna S., Irefin S., Mousa A., Maheshwari K. (2024). Intraoperative hypotension during liver transplantation and postoperative outcomes: Retrospective cohort study. J. Clin. Anesth..

[32] Cabezuelo J.B., Ramírez P., Ríos A., Acosta F., Torres D., Sansano T., Pons J.A., Bru M., Montoya M., Bueno F.S. (2006). Risk factors of acute renal failure after liver transplantation. Kidney Int..

[33] Joosten A., Lucidi V., Ickx B., Van Obbergh L., Germanova D., Berna A., Alexander B., Desebbe O., Carrier F.M., Cherqui D. (2021). Intraoperative hypotension during liver transplant surgery is associated with postoperative acute kidney injury: A historical cohort study. BMC Anesthesiol..

[34] Guo M., Gao Y., Wang L., Zhang H., Liu X., Zhang H. (2020). Early acute kidney injury associated with liver transplantation: A retrospective case-control study. Med. Sci. Monit..

[35] Cai L., Shu L., Yujun Z., Ke C., Qiang W. (2023). Lack of furosemide responsiveness predicts severe acute kidney injury after liver transplantation. Sci. Rep..

[36] Wu Z., Wang Y., He L., Jin B., Yao Q., Li G., Wang X., Ma Y. (2023). Development of a nomogram for the prediction of acute kidney injury after liver transplantation: A model based on clinical parameters and postoperative cystatin C level. Ann. Med..

[37] Catalán R., Jiménez-Ceja J.V., Rincón-Pedrero R., Olivas-Martínez A., Martínez-Rueda A.J., Bazúa-Valenti S., Carrillo-Pérez D.L., Grajeda-Medina L.I., García-Juárez I., Vilatobá M. (2022). Factors Associated with Development of Acute Kidney Injury After Liver Transplantation. Rev. Investig. Clin..

[38] Tan L., Yang Y., Ma G., Zhu T., Yang J., Liu H., Zhang W. (2019). Early acute kidney injury after liver transplantation in patients with normal preoperative renal function. Clin. Res. Hepatol. Gastroenterol..

[39] Ren A., Li Z., Zhang X., Deng R., Ma Y. (2020). Optimal timing of initiating CRRT in patients with acute kidney injury after liver transplantation. Ann. Transl. Med..

[40] Gao Q., Zhu L., Liu A.J., Gao J., Kong X.Y., Luan J.P., Cai J.Z., Dong H. (2025). The association between potassium level during the anhepatic stage of orthotopic liver transplantation and postoperative acute kidney injury: An exploratory study. BMC Nephrol..

[41] Tahir M., Nawaz H., Iqbal A., Safdar H., Naeem M., Ahmed A., Jamil M.I., Ghani F., Memon M.A., Afzal M. (2025). Incidence and clinical outcomes of postoperative acute kidney injury in relation to etiological factors and surgical procedures. Cureus.

[42] Schroeder R.A., Collins B.H., Tuttle-Newhall E., Robertson K., Plotkin J., Johnson L.B., Kuo P.C. (2004). Intraoperative fluid management during orthotopic liver transplantation. J. Cardiothorac. Vasc. Anesth..

[43] Jung J.Y., Ju J.W., Yoon H.K., Lee H.J., Kim W.H. (2024). Intraoperative normal saline administration and acute kidney injury in patients undergoing liver transplantation. Transplant. Proc..

[44] Hand W.R., Whiteley J.R., Epperson T.I., Tam L., Crego H., Wolf B., Chavin K.D., Taber D.J. (2015). Hydroxyethyl starch and acute kidney injury in orthotopic liver transplantation: A single-center retrospective review. Anesth. Analg..

[45] Mukhtar A., Aboulfetouh F., Obayah G., Salah M., Emam M., Khater Y., Akram R., Hoballah A., Bahaa M., Elmeteini M. (2009). The safety of modern hydroxyethyl starch in living donor liver transplantation: A comparison with human albumin. Anesth. Analg..

[46] Hannon V., Kothari R.P., Zhang L., Bokoch M.P., Hill R., Roll G.R., Mello A., Feiner J.R., Liu K.D., Niemann C.U. (2020). The association between vena cava implantation technique and acute kidney injury after liver transplantation. Transplantation.

[47] Della Rocca G., Pompei L., Costa M.G., Coccia C., Scudeller L., Di Marco P., Monaco S., Pietropaoli P. (2004). Fenoldopam mesylate and renal function in patients undergoing liver transplantation: A randomized, controlled pilot trial. Anesth. Analg..

[48] Biancofiore G., Della Rocca G., Bindi L., Romanelli A., Esposito M., Meacci L., Urbani L., Filipponi F., Mosca F. (2004). Use of fenoldopam to control renal dysfunction early after liver transplantation. Liver Transpl..

[49] Kandil M.A., Abouelenain K.M., Alsebaey A., Rashed H.S., Afifi M.H., Mahmoud M.A., Yassen K.A. (2017). Impact of terlipressin infusion during and after live donor liver transplantation on incidence of acute kidney injury and neutrophil gelatinase-associated lipocalin serum levels: A randomized controlled trial. Clin. Transplant..

[50] Kwon H.M., Kang S.J., Han S.B., Kim J.H., Kim S.H., Jun I.G., Song J.G., Hwang G.S. (2024). Effect of dexmedetomidine on the incidence of postoperative acute kidney injury in living donor liver transplantation recipients: A randomized controlled trial. Int. J. Surg..

[51] Weinberg L., Broad J., Pillai P., Chen G., Nguyen M., Eastwood G.M., Scurrah N., Nikfarjam M., Story D., McNicol L. (2016). Sodium bicarbonate infusion in patients undergoing orthotopic liver transplantation: A single center randomized controlled pilot trial. Clin. Transplant..

[52] Emara M.M., Diab D.G., Yassen A.M., Abo-Zeid M.A. (2022). Mannitol for prevention of acute kidney injury after liver transplantation: A randomized controlled trial. BMC Anesthesiol..

[53] Brescia M.D., Massarollo P.C., Imakuma E.S., Mies S. (2015). Prospective randomized trial comparing hepatic venous outflow and renal function after conventional versus piggyback liver transplantation. PLoS ONE.

[54] Perilli V., Aceto P., Sacco T., Modesti C., Ciocchetti P., Vitale F., Russo A., Fasano G., Dottorelli A., Sollazzi L. (2016). Anaesthesiological strategies to improve outcome in liver transplantation recipients. Eur. Rev. Med. Pharmacol. Sci..

[55] Giglio M., Dalfino L., Puntillo F., Brienza N. (2019). Hemodynamic goal-directed therapy and postoperative kidney injury: An updated meta-analysis with trial sequential analysis. Crit. Care.

[56] Punzo G., Di Franco V., Perilli V., Sacco T., Sollazzi L., Aceto P. (2023). Efficacy and safety of prothrombin complex concentrates in liver transplantation: Evidence from observational studies. J. Clin. Med..

[57] Teofili L., Valentini C.G., Aceto P., Bartolo M., Sollazzi L., Agnes S., Gaspari R., Avolio A.W. (2022). High intraoperative blood product requirements in liver transplantation: Risk factors and impact on the outcome. Eur. Rev. Med. Pharmacol. Sci..

[58] Aceto P., Punzo G., Di Franco V., Teofili L., Gaspari R., Avolio A.W., Del Tedesco F., Posa D., Lai C., Sollazzi L. (2023). Viscoelastic versus conventional coagulation tests to reduce blood product transfusion in patients undergoing liver transplantation. Eur. J. Anaesthesiol..

[59] Skytte Larsson J., Bragadottir G., Redfors B., Ricksten S.E. (2018). Renal effects of norepinephrine-induced variations in mean arterial pressure after liver transplantation: A randomized cross-over trial. Acta Anaesthesiol. Scand..

[60] Jan M.Y., Patidar K.R., Ghabril M.S., Kubal C.A. (2025). Optimization and protection of kidney health in liver transplant recipients: Intra- and postoperative approaches. Transplantation.

