# Supplementary material for: Can Intraoperative Anesthesiological Management Reduce the Risk of Acute Kidney Injury After Liver Transplantation? A Systematic Review
Source: J Clin Med. 2026 Mar 12;15(6):2181. doi: 10.3390/jcm15062181 (PMC13026614; doi:10.3390/jcm15062181)
Supplement: Supplementary file 1 [file jcm-15-02181-s001.zip › jcm-4141766-supplementary.pdf]

Appendix 1

Detailed Search Strategy (January 2004-November 16, 2025)

1. Cochrane CENTRAL search strategy through Cochrane Library (https://www.cochranelibrary.com/advanced-search/mesh)

("acute kidney injury" OR AKI) AND ("liver transplantation" OR "liver transplant*") AND (intraoperative OR intraoperatively OR intra-op)

2. Pubmed/MEDLINE search strategy

("acute kidney injury"[MeSH Terms] OR ("acute"[All Fields] AND "kidney"[All Fields] AND "injury"[All Fields]) OR "acute kidney injury"[All Fields]) AND ("liver transplantation"[MeSH Terms] OR ("liver"[All Fields] AND "transplantation"[All Fields]) OR "liver transplantation"[All Fields]) AND ("intraop"[All Fields] OR "intraoperative"[All Fields] OR "intraoperatively"[All Fields])

2. EMBASE search strategy

('acute kidney injury' OR AKI) AND ('liver transplantation' OR 'liver transplant*') AND (intraoperative OR intraoperatively OR intra-op)

4. Scopus Database search strategy

("acute kidney injury" OR AKI) AND ("liver transplantation" OR "liver transplant*") AND (intraoperative OR intraoperatively OR intra-op)

Table S1. Characteristics of studies included.

Study, Year	Study Design	Sample, n	Donor, DCD, DBD or L (%)	AKI Classification	Prevalence of AKI	Primary Outcome	Other Outcome	Main Results Related to IO Risk Factors of AKI
**Berkowitz R.J. et al., 2021 [2]**	Retrospective	598	DCD (6.7%) / DBD (89.6%)	KDIGO	43%	Stage 2 or 3 AKI within the first 7 postoperative days.	Survival	Independent predictors: increased potassium value during reperfusion and lactate during reperfusion.
**Kim W.H. et al., 2018 [14]**	Retrospective	734	L	KDIGO	36.1%	Increase of SCr (stage 2 or 3 of KDIGO guidelines) within the first 7 days after LT.	PO RRT, ICU LoS, hospital LoS, and in-hospital and 1-year mortality.	The elevated baseline CVP, elevated baseline RVEDV after anesthesia induction, and decreased SvO2 during the anhepatic phase were associated with PO AKI.
**Emara M.M. et al., 2022 [52]**	RCT	84	L	ICA	23.8%	A 0.3 mg/dl increase in the SCr in the early 48 PO hours.	PRS, early PO graft function, and 3-month survival.	1 g/kg mannitol infusion did not reduce the risk of early AKI or PRS or have any effect on other outcomes.
**Caragata R. et al., 2023 [3]**	Retrospective	1292	L (25.8%)/ DBD (64.0%)/ DCD (10.1%)	KDIGO	40%	Any AKI stages during the first two postoperative days	Long-term effects of AKI on renal function: the highest sCr at 30 ± 5 days, sCr at 1 year ± 1 month, and the need for RRT during the transplant admission.	Prolonged (>20 min) IOH (< 55 mmHg) was independently associated with AKI following LT, after adjusting for several known confounders.
**Zongyi Y. et al., 2016 [5]**	Retrospective	4482	DCD	RIFLE	3.97%	AKI within the hospital stay period.	1- and 5-year CRS	Independent risk factors: blood loss, cold ischemia time, warm ischemia time.
**Sirivatanauksorn Y. et al., 2014 [4]**	Retrospective	81	NS	Increased SCr level more than 1.5 times	71.6%	ARF	Long-term renal dysfunction was characterized by	An independent predictor was IOH (MAP< 70 mmHg) for

				within the first week after LT versus preoperative level.			serum creatinine levels more than 2.0 mg/dL after 1 year of ARF diagnosis. ICU stay, in-hospital mortality.	more than 30 minutes. During the IO and PO period, the ARF group required more blood component transfusions.
**Zhang D. et al., 2021 [15]**	Retrospective	204	DCD	KDIGO	55.4%	AKI in the first week after LT	Renal function and mortality rate 4 weeks after LT.	Risk factors for AKI were cold ischaemia time, duration of surgery, duration of inferior vena cava clamping.
**Han Y.Z. et al., 2021 [10]**	Retrospective	115	DCD	KDIGO	38.3%	The incidence of new AKI within 72 hours after the operation.	28-day mortality, length of ICU and hospital stay, and incidence of EAD and PNF.	The levels of serum albumin, alanine transaminase, and Mb on admission to the ICU were independently associated with the development of new AKI.
**Barreto A.G.C. et al., 2015 [6]**	Retrospective	134	NS	AKIN	46.7%	AKIN as an increase of more than two times in S. Cr (AKIN 2 or 3) in the first 72 h after LT. The diagnosis of AKI was based on only one of the components of the AKIN, since data on urinary output were not available for all patients.	ICU stay, hospitalization, mortality, CKD (GFR < 60 mL/min/1.73 m2 three months after AKI episode)	Risk factors for AKI included longer warm ischemia time and higher serum lactate levels.
**Yoo S. et al, 2017[11]**	Retrospective	304	DBD/L	RIFLE	43.4%	PO AKI (up to POD7)	Persistent renal dysfunction (RIFLE criteria) up to 6 months	Increased perioperative glucose variability, measured by standard deviation of IO and PO (initial 48 hours of ICU admission) blood glucose levels, but not hyperglycemia, is independently associated with an increased risk of PO AKI in LT recipients.
**Hannon V. et al., 2022 [46]**	Retrospective	897	DBD	ICA	40.3%(Piggyback) to 51.8%	AKI stage ≥2. Baseline S.Cr was defined as the lowest level within 48 h	Kidney function at 1 week, discharge and at 1 year after LT.	Reduced risk of higher-stage AKI using the piggyback technique

					(caval replacement)	before surgery. Peak S. Cr was defined as the highest creatinine within 48 h after surgery.	Renal function (estimated glomerular filtration rate and the number of patients alive without dialysis one y after transplant.	compared with caval replacement. The warm ischemia time was shorter in the piggyback group and was identified as a potential mediator of this effect.
**Wyssusek K.H. et al., 2015 [16]**	Retrospective	97	DBD	SCr >133 μmol/l with an increase of 50% above the baseline and/or the requirement for RRT.	25%	AKI in the first week following LT.	Overall mortality	An independent risk factor was the use of IO noradrenaline.
**Guo D. et al., 2021 [17]**	Retrospective	576	NS	KDIGO	34.3% and 40.5% (development and validation cohort, respectively)	AKI (48 h or 7 days after LT)<br>The maximum S. Cr level was recorded during the first 7 days after surgery and compared to the baseline level.	Long-term mortality and development of CKD.<br>The follow-up period was 29.5 months (interquartile range: 16-52 months)	An independent risk factor was IO FFP transfusion for post-LT severe AKI.
**Hand W.R. et al., 2015 [44]**	Retrospective	174	NS	RIFLE	14-38%	AKI was staged into Risk, Injury and Failure based on the change in S. Cr from the preoperative baseline to the peak level by PO day 7.	PO outcomes (HAT, PVT, ICU-LOS, hospital LOS, Observed direct costs, IO death, 30-day mortality).	Patients receiving 6% HES (130/0.4) likely had increased odds of AKI compared with patients receiving 5% albumin during LT.
**Hilmi I.A. et al., 2015 [18]**	Retrospective	221	DBD	KDIGO	52%	AKI within 72 h.	Incidence of new CKD and requirement for dialysis at 3 months and 1 year post-transplant.<br>Chronic kidney disease (CKD) was defined according to the criteria established by KDOQI.	Transfusion requirements were an independent predictor of AKI, even after adjusting for hemodynamic instability.

**Nadeem A., 2014 [19]**	Retrospective	158	L/DBD	RIFLE	36.1%	AKI up to the third day.	Delayed weaning from MV, prolonged ICU stay, ICU mortality, and 28-day mortality.	Infusion of higher volumes of chloride-liberal fluids and the preoperative status was associated with an increased risk for PO AKI.
**Zhou Z.B. et al., 2015 [20]**	Retrospective	394	NS	PO renal impairment = 1.5-mg/dL increase in the S Cr in patients previously having a normal renal function or	35%	PO renal impairment during the first PO week.	NS	An independent predictor was the use of massive red blood cell transfusions. The perioperative use of HES 200/0.5 or HES 130/0.4 did not significantly affect renal function during the first PO week.
**Mizota T. et al., 2017 [21]**	Retrospective	231	L	KDIGO	30.7%	Severe AKI, defined as stage 2-3 AKI according to the guidelines of KDIGO up to 7th PO Day.	NS	Independent relationship between the degree of IOH and risk of severe AKI. Severe hypotension, even for a short duration, was significantly related to severe AKI.
**Widmer J.D. et al., 2018 [22]**	Retrospective	586	DBD	KDIGO	14.8%	AKI within the first 7 days after transplantation.	Complications, including PNF and HAT	Both liver implantation techniques, piggy-back or cava replacement, are equal in terms of kidney function and overall complications following LT.
**Zhang S. et al., 2020 [12]**	Retrospective	146	DCD	KDIGO	50%	AKI within 3 days after LT.	Complications including EAD, biliary tract complications, pulmonary infection, vascular complications, hepatocellular carcinoma recurrence, and acute rejection. Complication-free survival.	Cumulative FB within 72 hours was associated with post-OLT AKI and required RRT.

**Carrier F.M. et al., 2020 [9]**	Prospective	532	DCD/ DBD/ L	KDIGO	AKI (stage 2-3) = 57.6% at 48 hours and 24.7% at 7 days. All stages AKI = 69.8% at 48 hours and 32.6% at 7 days.	48-hour and 7-day AKI	7-day AKI, need for PO RRT, time to extubation in the ICU, time to ICU discharge, and survival up to 1 year.	The presence of any vasopressor at the end of surgery was not associated with the development of AKI or the need for RRT. A higher FB was associated with a higher need for RRT only among patients without vasopressors.
**Schroeder R.A. et al., 2024 [42]**	Retrospective	151	DBD	Peak creatinine Need for RRT	NS	S Cr during hospitalization until peak (first hours/days after OLT).<br>Need for RRT: assessed in the immediate PO period	ICU and hospital LoS and 30-day mortality	Low CVP increased PO creatinine peak and dialysis
**Chen J. et al., 2011 [7]**	Retrospective	334	DBD/D CD	RIFLE	38.4%	ARI/ARF within the first week post-LT	Morbidity, reoperation, RRT, hospital LOS, graft survival, mortality	Post-LT ARI or ARF was associated with IO RBC transfusions and vasopressor use.
**Leithead J.A. et al., 2013 [25]**	Retrospective	296	DBD	RIFLE	35.9%	Following transplantation but before hospital discharge	NS	Independent predictors were PRBC transfusions (≥5 units) and the use of inotropes.
**Wang Y. et al., 2018 [26]**	Retrospective	137	DCD	KDIGO	51.8%	AKI within 7 days of surgery	Incidence of severe AKI (KDIGO stage ≥2 or patients requiring RRT), prolonged ICU stay<br>(PO stay in ICU >7 days), hospital LOS, and in-hospital death.	Transfusion of older RBCs (that had been stored for more than 14 days) significantly increased the risk of PO AKI in liver transplant recipients.
**Kandil M.A. et al., [49]**	RCT	50	L	ICA and ADQI criteria	44-48%	AKI within 48 h	Serum concentration of NGAL (2 and 24 h after reperfusion)	Post-liver transplant AKI was not prevented by terlipressin use nor predicted by NGAL levels.
**Brescia M.D.et al., 2015 [53]**	RCT	32	DBD	RIFLE-AKIN	Overall ARF (Class R + I + F) was 79% within 48 hours, 86%	Free hepatic vein pressure (FHVP) and CVP measurements were performed after graft reperfusion.	The prevalence of ARF up to the 28th PO.	Conventional with venovenous bypass technique significantly increases the harm of PO renal dysfunction.

					within the first week and 90% at the 28th PO			
**Mukhtar A. et al., 2009 [45]**	RCT	40	L	ARF requiring dialysis	NS	Renal function (S Cr at the end of surgery, and on the first 4 days postoperatively + discharge from the hospital); CrCl was measured on PO Days 1, 3, and 5, and at discharge.	NS	The use of HES 130/0.4 as an alternative to human albumin resulted in equivalent renal outcome after LT.
**Weinberg L. et al., 2016 [51]**	RCT	60	NS	RIFLE	34%	AKI (RIFLE Class "R") within the first 48 h post-operatively.	Complications, ICU LOS, hospital LOS	The intra-operative infusion of sodium bicarbonate did not decrease the incidence of AKI in patients following LT.
**Fiorelli S. et al. 2022 [1]**	Retrospective	1681	NS	RIFLE	Overall AKI 21.9%	Early PO AKI incidence during the first 72 h after OLT	perioperative risk factors, and AKI impact on survival	IO risk factors for AKI were: administration of 5-10 RBCs, dopamine use, post-reperfusion syndrome, surgical complications, and cardiologic complications.
**Kwon H.M. et al., 2024 [50]**	RCT	205	L	KDIGO	42.4% overall (35% DEX vs 50% control, p=0.042)	Incidence of PO AKI	Serial IO lactate levels, CKD incidence, MACE, early allograft dysfunction, graft failure, mortality, ventilation duration, ICU/hospital LoS	Dexmedetomidine significantly reduced AKI incidence and IO lactate levels.
**Jung J.Y. et al., 2024 [43]**	Retrospective (with propensity score matching)	1011	L	KDIGO	Saline group (n = 88/174, 50.6%) vs balanced group (n = 67/174, 38.5%) after matching	Incidence of PO AKI (all stages, and stages 2–3)	Length of ICU/hospital stay, early allograft dysfunction, postoperative hemodialysis, in-hospital and 1-year	Normal saline administration during LDLT is associated with a higher risk of PO AKI, stage 2 or 3 AKI, and a longer hospital stay compared with balanced crystalloids.

					(p= .010)		mortality, graft failure	
**Chen C. et al., 2025 [24]**	Prospective (with machine-learning–based predictive modeling)	405	DBD, DCD	KDIGO Criteria (Stage 3 AKI= severe AKI)	Severe AKI: 10.8% (44/405)	Development of severe (Stage 3) PO AKI	Continuous RRT requirement, 14-day mortality, postoperative coagulation variables (TEG-R, D-dimer), model performance “SALT" graded nomograms for AKI prediction	The SALT I–III nomograms achieved AUROC values of 0.751–0.894 and incorporated EBL.
**Trung N.D. et al., 2025 [27]**	Retrospective	97	L	KDIGO	63.9% (Stage 1: 43.3%, Stage 2: 9.3%, Stage 3: 11.3%)	Incidence and severity of early PO AKI (within 7 days)	Risk factors (MELD, ischemia times, blood loss, lactate), ICU stay, hospital stay, RRT, 1-year mortality	Cold ischemia time, IO blood loss volume were identified as independent risk factors for early AKI.
**Antonucci E. et al., 2025 [28]**	Retrospective	1120	DBD 65.9%, DCD 10.4%, L 21.5%	ICA	Severe AKI (Stage ≥2) 29.2%; overall AKI any stage 67.1%	Severe AKI (Stage ≥2) within 48 h after transplantation	AKI requiring RRT, eGFR at 48 h, 1 wk, and 1 y; hospital LOS; mortality; graft survival	No association between the IO use of vasopressin and severe AKI.
**Bieze M. et al., 2024 [29]**	Retrospective	1153	DBD 55.5%, DCD 8.6%, L 35.9%	KDIGO criteria (S Cr only)	47.2% overall (Stage 1: 65.4%, Stage 2: 22.6%, Stage 3: 11.9%)	Association between IOH (MAP < thresholds) and postoperative AKI	Effect of anhepatic vs neohepatic phase hypotension, ischemia times, transfusions, donor type, and mortality	IOH independently associated with AKI. MAP <60 mmHg and MAP <55 mmHg durations > 20 min significantly increased AKI risk (adjusted OR 1.97 and 3.44, $p < 0.02$). Association is strongest during the anhepatic phase; the neohepatic phase is less predictive. AKI prevalence is more frequent in DCD grafts, longer CIT, and higher blood loss.
**Della Rocca G., 2004 [47]**	RCT	43	DBD	S. Cr and BUN or urine output	NS	S. Cr 1.4 mg/dL and BUN 22 mg/dL or urine output 800 mL/24 h or 200 mL/6 h within 72 h of surgery.	ICU LoS, Hospital LoS	Compared with dopamine, in the setting of liver transplantation, fenoldopam is associated

								with better CRE and BUN values.
**Biancofiore G, 2004 [48]**	RCT	140	DBD	S Cr ≥ 1.5 mg/dl, the doubling of serum creatinine within 24hours, or use of continuous RRT	10.7%	Renal dysfunction up to 96 h after the end of LT	ICU stay and mortality, hospital and mortality	CrCl in the patients receiving fenoldopam remained substantially unchanged, whereas it decreased by 39 and 12.3%, respectively, in the subjects receiving placebo or dopamine
**Mrzljak A., 2020 [30]**	Retrospective	205	DBD	KDIGO	45.36%	AKI (1 to 3 stages) within the first 7 post-operative days	30-d survival	Independent predictors: EBL, FFP, total fluid intake, surgery duration
**Cywinski J.B., 2004 [31]**	Retrospective	1576	DBD, DCD, L	KDIGO	73.6%	AKI stage (1 to 3) at 48 h, 7 days, and 30 days.	ICU LoS, hospital LoS, graft failure (1-3 years), mortality (1-3 years)	No significant link was found between hypotension exposure and postoperative AKI. The duration or intensity of MAP <55 mmHg did not increase the risk of or severity of AKI.
**Cabezuelo J.B., 2006 [32]**	Retrospective	200	DBD	ARF was defined as a 50% or greater increase in postoperative S-Cr compared to pre-LT values	48%	Overall ARF, early ARF (first week post-OLT) and late ARF (second to fourth week post-LT)	Mortality at 30 days	Univariate analysis revealed that the use of FFP, cryoprecipitate, noradrenaline, and dopamine was associated with AKI.
**Joosten A. et al., 2021 [33]**	Retrospective	205	DBD	KDIGO	57%	AKI (stages 1-3) at 48 h and up to the first PO week	NS	IOH was independently significantly associated with a higher risk of AKI. Each 1% increase in surgery time with MAP ≤65 mmHg raised AKI risk by approximately 5%. From univariate analysis: total intake, EBL, PRBC,

								and vasopressor use were associated with AKI.
**Guo M. et al., 2020 [34]**	Case control	122	NS	KDIGO	42.6%	AKI stages within 48 h	RRT, ICU LoS, hospital LoS, death, hospitalization expense	Factors associated with AKI: Operative time, total fluid intake, FFP, and EBL. Independent predictor: FFP.
**Cai L. et al., 2023 [35]**	Retrospective	214	DCD, DBD	KDIGO	49%	AKI stages within 48 h	6-month survival	Risk factor: increased IO blood loss.
**Wu Z.et al., 2023 [36]**	Prospective	120	NS	KDIGO	48.3%	AKI stages within 7 days	EAD, ARDS, duration of mechanical ventilation, shock, infection, RRT, ICU LoS, hospital mortality	Risk factors: blood product transfusions, prolonged an-hepatic phase.
**Catalàn R. et al., 2022 [37]**	Retrospective	86	NS	KDIGO	39.5%	AKI stages within 30 days	30-day mortality	High lactate concentration during surgery.
**Tan L. et al., 2019 [38]**	Retrospective	227	DCD, L	KDIGO	46.6%	AKI stages within 7 days	Ventilation time, ICU LoS, total hospitalization duration, postoperative nosocomial infection, reoperation, acute rejection.	AKI was linked to greater transfusion needs, higher IO blood loss (>10 units), and use of the caval replacement technique.
**Ren A. et al., 2020 [39]**	Retrospective	173	DCD, DBD	KDIGO	27.7%	Overall AKI and continuous RRT (early if within 24h and late if after 24h)	ICU LoS, hospital LoS, 30-day and 90-day mortality; PNF, EAD, Acute rejection.	EBL and plasma transfusion volumes were independent predictors of AKI. PRCB, platelets, and cryoprecipitate transfusions were associated factors.
**Gao Q. et al., 2025 [40]**	Retrospective	136	DCD	KDIGO	44.12%	AKI and serious AKI (up to 5th day)	One-year survival	Elevated anhepatic potassium levels were identified as an independent and strong predictor of postoperative AKI in multivariate analysis. Optimal potassium cutoff = 4.05 mmol/L.

**Tahir M. et al, 2025 [41]**	Prospective	175	NS	KDIGO	18.9%	Development and severity of PO AKI (stages 1-3), defined by increases in SCr within 48 hours or 7 days	Secondary outcomes included renal recovery status, need for RRT, ICU admission, and in-hospital mortality (during hospitalization and up to 30 days)	AKI was strongly associated with longer surgery, greater blood loss, and intraoperative transfusion, identifying surgical duration and bleeding as key intraoperative predictors.

Y, yes; N no; D, Deceased; L, living; DCD, donation after circulatory death; DBD, donation after brain death; RRT, renal replacement treatment; KIDCO, Kidney Disease Improving Global Outcomes; ICA, International Club of Ascites; ADQI, Acute Disease Quality Initiative; LoS, length of stay; ICU, intensive care unit; LT, liver transplantation; CVP, central venous pressure; RVEDV, right ventricular end-diastolic volume; SvO2, mixed venous oxygen saturation; RCT, randomized controlled trial; S.Cr, serum creatinine; NS, not stated; PRS, post-reperfusion syndrome; CRS, cumulative survival rate; EAD, early allograft dysfunction; Mb, myoglobin; FFP, fresh frozen plasma; PNF, primary allograft non-function; GRF, Glomerular filtration rate; CKD, Chronic kidney disease; RIFLE, risk, injury, failure, loss, and end-stage kidney disease; POD, postoperative day; AKI, acute kidney injury; SR, systematic review; MV, mechanical ventilation; WIT, warm ischaemia time; KDOQI, National Kidney Foundation's Kidney Disease Outcomes Quality Initiative; MELD, Model for End-stage Liver Disease; BMI, body mass index; HAT, Hepatic artery thrombosis; PVT, Portal vein thrombosis; CKD, chronic kidney disease; HES, hydroxyethyl starch; PNF, primary non function; RRT, renal replacement therapy; AKIN, Acute Kidney Injury Network; MAP, mean arterial pressure; GFR, glomerular filtration rate; NA, not applicable; FB, fluid balance; EAD, early graft dysfunction; ARI, acute renal injury; ARF, acute renal failure; AST; aminotransferase; NGAL, Neutrophil Gelatinase Associated Lipocalin; IO, intraoperative; PO, postoperative; CrCl, creatinine clearance; eGFR, estimated glomerular filtration rate; FHVP, Free hepatic vein pressure; CI, confidence interval; EBL, estimated blood loss; BUN, Blood Urea Nitrogen; PNF, primary nonfunction; EAD, early allograft dysfunction, MACE, Major adverse cardiac events; MAKE, Major adverse kidney events; ARDS, Acute respiratory distress syndrome. (*) The ICA adapts KDIGO's general AKI criteria to the specific physiology of patients with cirrhosis, whereas ADQI offers expert consensus frameworks that often build on or extend KDIGO but are not identical to it.

Table S2. Risk of bias table for assessing the quality of cohort studies by using the Newcastle-Ottawa Scale.

Study	Representativenes of the Exposed Cohort	Selection of the non Exposed Cohort	Ascertainment of Exposure	Demonstration that Outcome of Interest was not Present at Start of Study	Comparability of Cohorts on the Basis of the Gender	Comparability of Cohorts on the Basis of the Age	Comparability of Cohorts on the Basis of the MELD Score	Assessment of Outcome	Was Follow-up Long Enough for Outcomes to Occur	Adequacy of Follow up of Cohorts
Antonucci E. et al., 2025 [28]	*	*	*	*	*	*	*	*	*	*
Nadeem A. et al., 2014 [19]	*	*	*	*	*	*	*	*	*	*
Barreto A.G.C. et al., 2015 [6]	*	*	*	*		*	*	*	*	*
Berkowitz R.J. et al., 2021 [2]	*	*	*	*	*	*	*	*	*	*
Bieze M. et al., 2024 [29]	*	*	*	*	*	*	*	*	*	*
Cabezuelo J.B. et al., 2006 [32]	*	*	*						*	*
Cai L.et al., 2023 [35]	*	*	*	*				*	*	*
Caragata R. et al., 2023 [3]	*	*	*	*	*	*	*	*	*	*
Carrier F.M. et al., 2020 [23]	*	*	*	*	*	*	*	*	*	*
Catalan R. et al., 2022 [37]	*	*	*	*				*	*	*
Chen C. et al., 2025 [24]	*	*	*	*	*	*	*	*	*	*
Chen J. et al., 2011 [7]	*	*	*	*	*	*	*	*	*	*
Cywinski J.B. et al., 2004 [31]	*	*	*						*	*
Fiorelli S. et al., 2022 [1]	*	*	*	*	*	*	*	*	*	*
Gao Q. et al., 2025 [40]	*	*	*	*				*	*	*
Guo D. et al., 2021 [17]	*	*	*	*	*	*	*	*	*	*
Guo M. et al., 2020 [34]	*	*	*	*	*	*	*	*	*	*
Han Y.-Z. et al., 2021 [10]	*	*	*	*				*		
Hand W.R. et al., 2015 [44]	*	*	*	*		*	*	*	*	*

Hannon V. et al., 2022 [46]	*	*	*	*	*	*	*	*	*	*
Hilmi I.A. et al., 2015 [18]	*	*	*	*	*	*	*	*	*	*
Joosten A. et al., 2021 [33]										
Jung J.Y. et al., 2024 [43]	*	*	*	*	*	*	*	*	*	*
Kim W.H. et al., 2018 [14]	*	*	*	*	*	*	*	*	*	*
Leithead J.A. et al., 2013 [25]	*	*	*	*	*	*	*	*	*	*
Mizota T. et al., 2017 [21]	*	*	*	*				*	*	*
Mrzljak A. et al., 2020 [30]	*	*	*	*				*	*	*
Ren A. et al., 2020 [39]	*	*	*	*				*	*	*
Schroeder R.A. et al., 2024 [42]	*		*						*	*
Sirivatanauksorn Y. et al., 2014 [4]		*	*	*					*	
Tahir M.et al., 2025 [41]	*	*	*	*	*	*	*	*	*	*
Tan L. et al., 2019 [38]	*	*	*	*				*	*	*
Trung N.D. et al., 2025 [27]	*	*	*	*	*	*	*	*	*	*
Wang Y. et al., 2018 [26]	*	*	*	*	*	*	*	*	*	*
Widmer J.D. et al., 2018 [22]	*	*	*	*	*	*		*	*	*
Wu Z. et al., 2023 [36]	*	*	*	*				*	*	*
Wyssusek K.H. et al., 2015 [16]		*	*	*			*	*	*	
Yoo S. et al., 2017 [11]	*	*	*	*	*	*	*	*	*	*
Zhang S. et al., 2020 [12]	*	*	*	*	*	*	*	*	*	*
Zhang D. et al., 2021 [15]	*	*	*	*	*	*		*	*	*
Zhou Z.B. et al., 2015 [20]	*	*	*	*					*	*
Zongyi Y. et al., 2016 [5]	*	*	*	*	*			*	*	*

* indicates low risk of bias/domain fulfilled. Blank = potential or unclear bias.
Criteria based on the Newcastle–Ottawa Scale: representativeness – consecutive or registry cohorts; selection – same-center non-exposed group; exposure – objective intraoperative data; outcome – KDIGO/RIFLE/AKIN definition; comparability – adjusted for gender, age, and MELD; follow-up – ≥ 7 days or hospital discharge with ≥ 90 % data completeness.

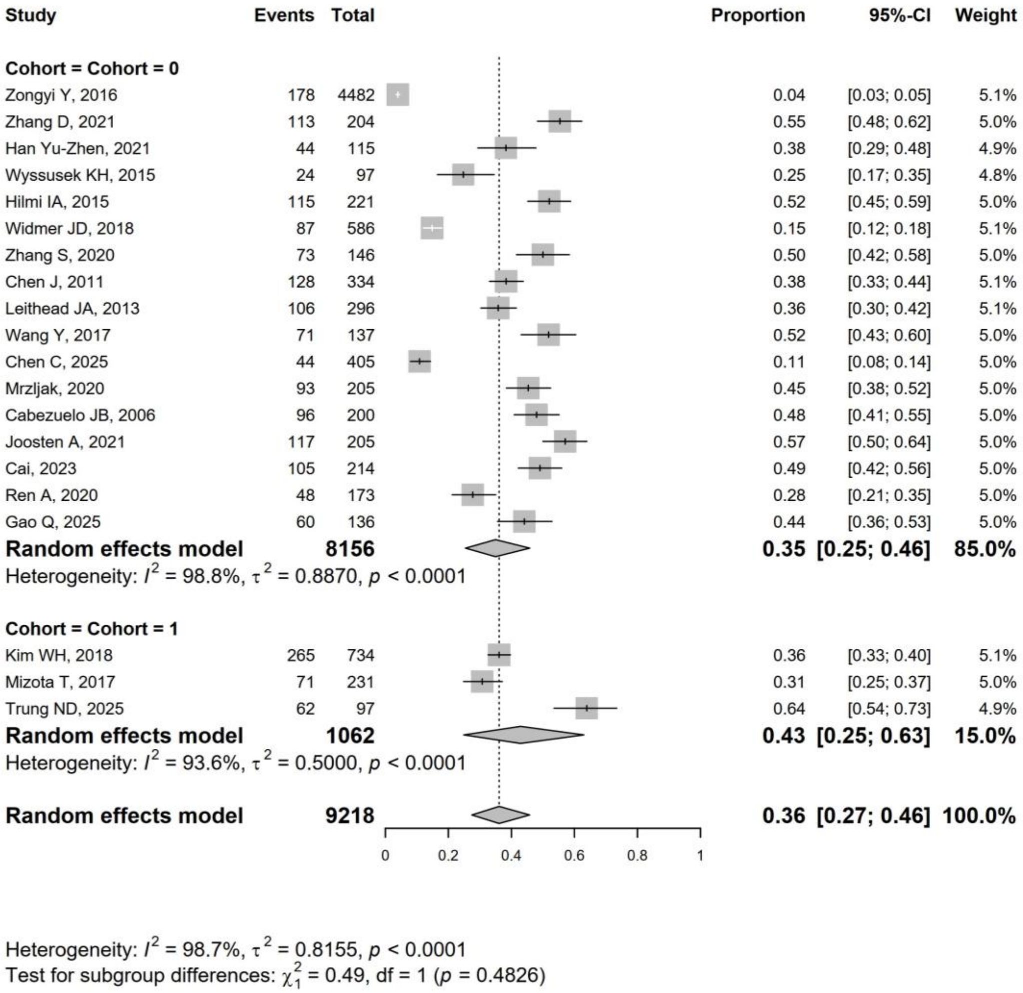

Figure S1. Forest plot of pooled AKI incidence by cohort 1=DDLT vs cohort 0=LDLT). Random-effects analysis showed no significant difference between cohorts (p = 0.48), despite substantial heterogeneity (I^2 = 98.7%).

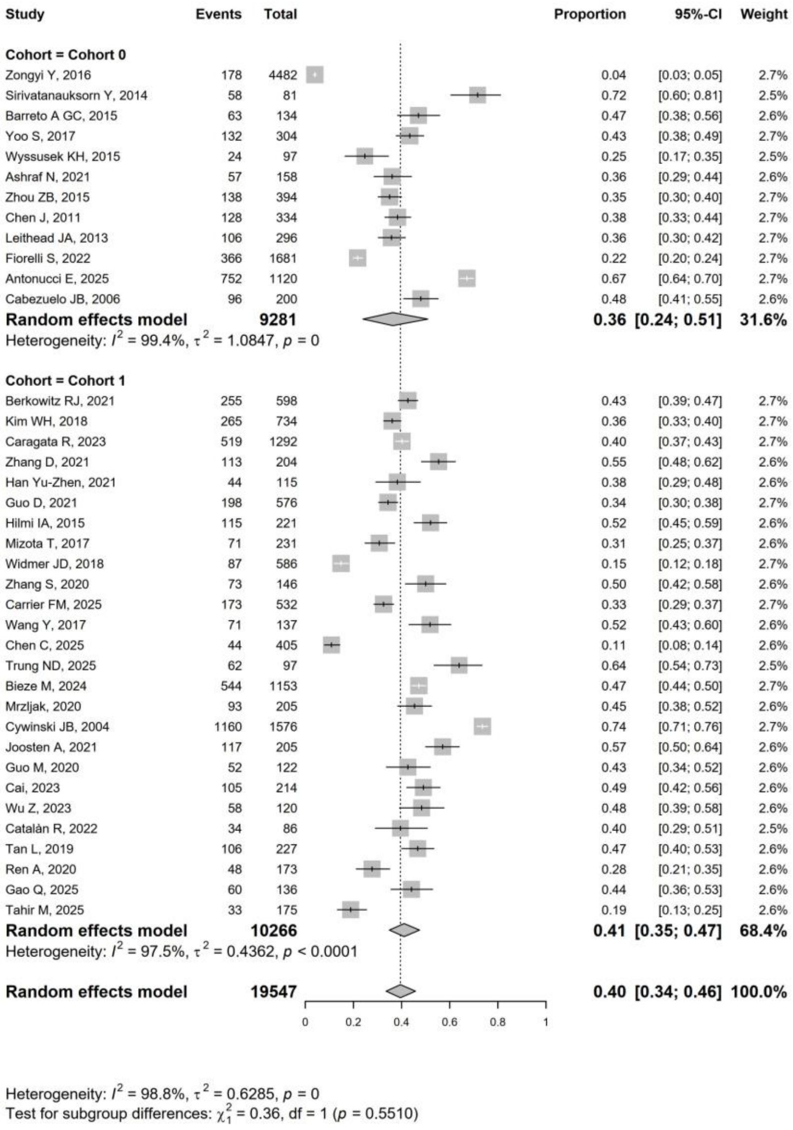

Figure S2. Forest plot of pooled AKI incidence by cohort 1=KDIGO vs cohort 0=no KDIGO). Random-effects meta-analysis of AKI incidence by cohort showing no significant difference between groups (p = 0.55) despite high heterogeneity (I^2 = 98.8%).

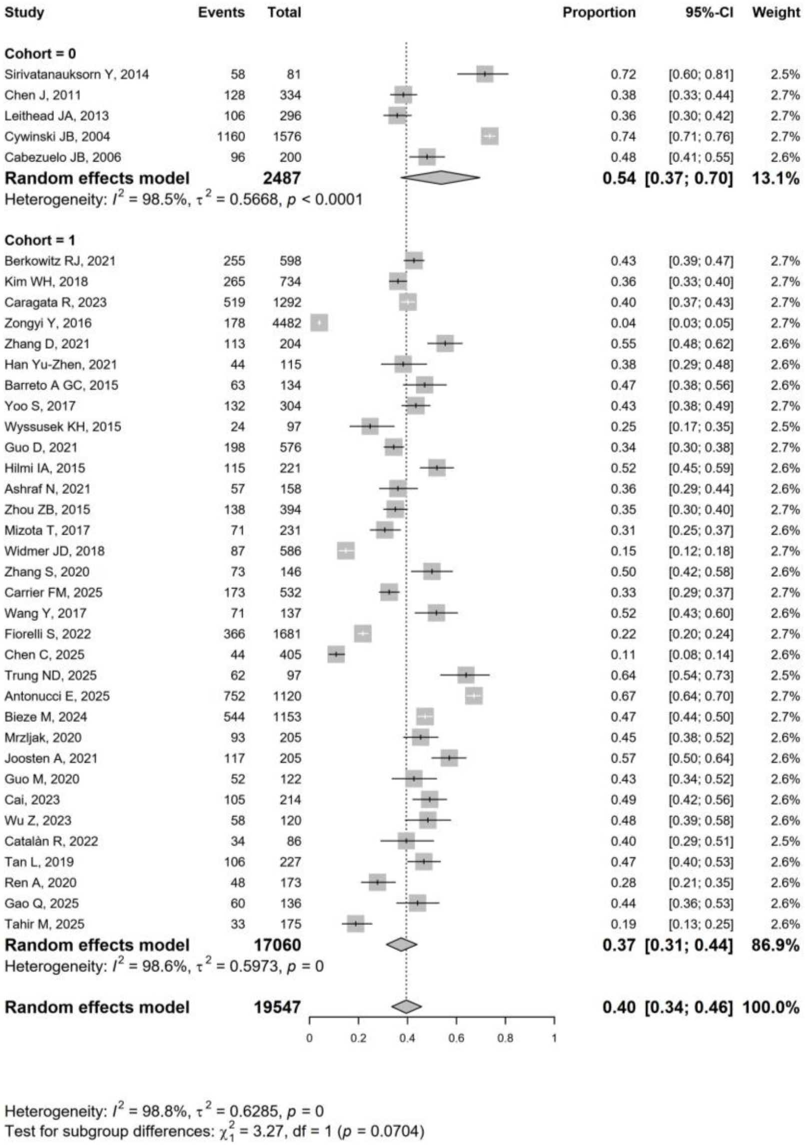

Figure S3. Forest plot of pooled AKI incidence by cohort 1=2015-2025 vs cohort 0=2004-2014. Random-effects meta-analysis of AKI incidence by cohort showing no significant difference between groups (p = 0.07) despite high heterogeneity (I^2 = 98.8%).

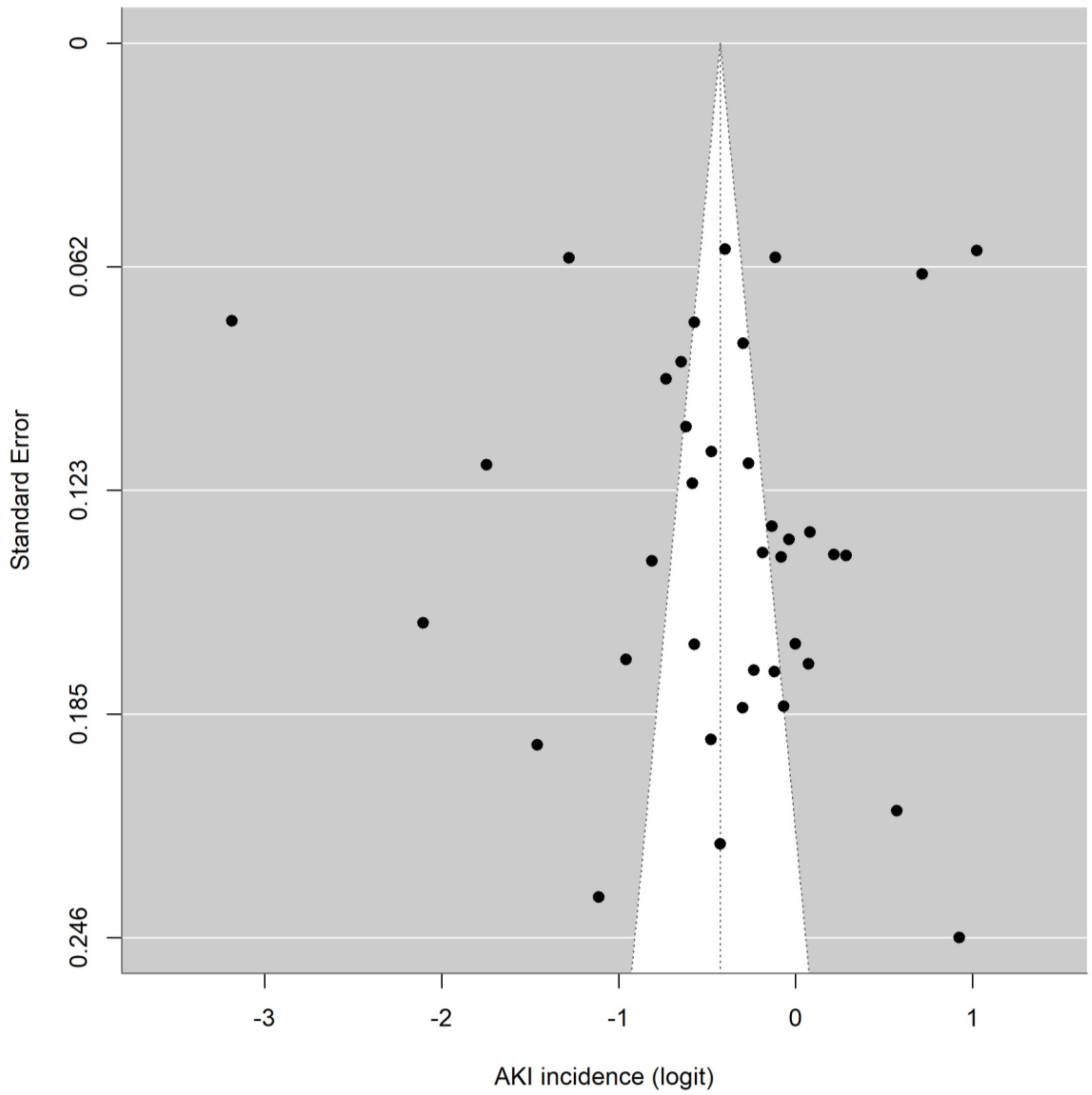

Figure S4. Funnel plot of AKI incidence (logit scale) showing no clear evidence of asymmetry, consistent with the absence of statistically significant small-study effects (Egger's test $p = 0.51$).
